# A Crucial Angiogenesis-Associated Gene MEOX2 Could Be a Promising Biomarker Candidate for Breast Cancer

**DOI:** 10.3389/fonc.2022.759300

**Published:** 2022-05-09

**Authors:** Huxia Wang, Yanan Tang, Xiaomin Yang, Weiyi Wang, Pihua Han, Jing Zhao, Sai He, Peijun Liu

**Affiliations:** ^1^ Center for Translational Medicine, The First Affiliated Hospital of Xi’an Jiaotong University, Xi’an, China; ^2^ Mammary Department, Shaanxi Provincial Cancer Hospital, Xi’an, China; ^3^ Vascular Surgery Department, The First Affiliated Hospital of Xi’an Jiaotong University, Xi’an, China

**Keywords:** angiogenesis, bioinformatics analysis, biomarker, breast cancer, MEOX2

## Abstract

**Background:**

Angiogenesis plays a critical role in the growth and metastasis of breast cancer and angiogenesis inhibition has become an effective strategy for cancer therapy. Our study aimed to clarify the key candidate genes and pathways related to breast cancer angiogenesis.

**Methods:**

Differentially expressed genes (DEGs) in the raw breast cancer (BRCA) gene dataset from the Cancer Genome Atlas (TCGA) database were identified and gene ontology analysis of the DEGs was performed. Hub genes were subsequently determined using the Gene Expression Omnibus database. The expression of the mesenchyme homeobox 2 (MEOX2) in breast cancer cells and tissues was assessed by quantification real-time polymerase chain reaction (qRT-PCR) and immunohistochemistry (IHC), respectively. The prognostic value of the MEOX2 gene in breast cancer tissue was evaluated with the Kaplan-Meier plotter.

**Results:**

A total of 61 angiogenesis-related DEGs were identified in the TCGA dataset, among which the gene MEOX2 was significantly down-regulated. GO functional annotation and pathway enrichment analyses showed that MEOX2 was significantly enriched in the regulation of vasculature development. The IHC results confirmed that MEOX2 expression was repressed in breast cancer tissues and the relatively low level indicated the tissue was densely vascularized. Moreover, MEOX2 expression was significantly elevated in breast cancer cells after treatment with cisplatin (DDP) and epirubicin (EPI). Finally, the Kaplan-Meier plotter confirmed that higher expression levels of MEOX2 were related to better overall survival.

**Conclusion:**

Our study revealed that the angiogenesis-associated gene MEOX2 can be used as a novel biomarker for breast cancer diagnosis and clinical therapy.

## Introduction

Breast cancer is the most common cancer among women and is the main cause of cancer-related deaths in women (1, 2). To date, the incidence of breast cancer continues to increase globally (3). A large amount of data shows that angiogenesis can significantly promote breast cancer growth and metastasis (4–6). Anti-angiogenic therapy has been used in breast cancer research; however, there remain a large number of patients who do not benefit from this treatment (7–9). Additionally, the role and mechanism of anti-angiogenic drugs in breast cancer are still unclear and further investigation is necessary. Hence, identifying new biomarkers of angiogenesis is essential for breast cancer diagnosis and therapy.

In recent years, the application of microarrays based on high-throughput sequencing technologies in cancer clinical research has provided an efficient tool for identifying biomarkers for cancer diagnosis and treatment (10–12). In the past few years, large gene microarray studies have been conducted to determine the carcinogenic effects of breast cancer and various differentially expressed genes (DEGs) have been identified. However, gene expression profiling analysis for breast cancer angiogenesis is still limited.

In the present study, the raw breast cancer (BRCA) dataset, which consists of data for 1102 breast cancer cases and 113 normal breast tissue samples, was obtained from the Cancer Genome Atlas (TCGA) database. We processed the raw data using packages in R software and analyzed DEGs using the DESeq2 package. The significantly down-regulated and angiogenesis-associated gene MEOX2 was identified. Afterward, the DAVID online database was applied for MEOX2 Gene ontology (GO) term enrichment analysis and Kyoto Encyclopedia of Genes and Genomes (KEGG) pathway analysis. The protein–protein interaction (PPI) network was constructed with STRING and visualized with Cytoscape software. Subsequently, the expression and function of MEOX2 were assessed based on the GEO dataset. We also investigated the expression of MEOX2 and CD31 in BRCA tissues. Finally, survival analysis for MEOX2 was performed on the Kaplan–Meier plotter website. Thus, we identified a crucial angiogenesis-associated gene MEOX2 in breast cancer.

## Materials and Methods

### Microarray Data

The BRCA dataset containing information for 113 normal and 1102 tumor samples was obtained from the TCGA Data Portal website (https://portal.gdc.cancer.gov). The gene expression profile for the GSE42568 dataset was obtained from the GEO database. A total of 104 breast cancer samples and 17 normal breast biopsies samples were evaluated in this array (Platforms: GPL570 Affymetrix Human Genome U133 Plus 2.0 Array). GSE119262, which consists of 21 pairs of breast cancer tissue samples treated with and without everolimus, is based on the GPL6104 Platform (Illumina humanRef-8 v2.0 expression bead chip). GSE55897 is based on the GPL10904 Platform (Illumina HumanHT-12 V4.0 expression bead chip) and contains different breast cancer paired cell samples treated with and without Indole-3-carbinol (I3C).

### Identification of DEGs

Our strategy consisted of first analyzing the related angiogenesis terms through GO enrichment analysis and then using the above terms to obtain the angiogenesis-associated DEGs. We analyzed the TCGA dataset using the TCGA biolinkers packages in R software and processed the downloaded data using dplyr in R software. After transforming the probe IDs into gene symbols, we performed background correction and log2 transformation. DEGs between breast cancer tissues and adjacent normal tissues were analyzed using the DESeq2 package in R language with the following criteria: adjusted *p* < 0.05 and |logFC| ≥ 1.

GEO datasets were analyzed using the limma package and the results were further processed in R software. The DEGs were selected according to the criteria: *p* < 0.05 and |logFC| ≥ 2.

### Gene Ontology Enrichment Analysis

DAVID (https://david.ncifcrf.gov/) is an online database for gene functional enrichment analysis (13). GO functional analysis was performed using DAVID and *p* values < 0.05 were considered significant.

### PPI Network Construction and Module Analysis

To assess functional interactions between MEOX2 and the associated proteins, we entered the DEGs into the STRING online database and constructed PPI networks using a score ≥ 0.4 as the cutoff threshold (14). The PPI networks were visualized using Cytoscape software and Molecular Complex Detection (MCODE) was applied to establish the module for MEOX2 (15).

### Immunohistochemistry Staining

Tissues were fixed in 10% neutralized formaldehyde for 24 h and then embedded in paraffin. The paraffin-embedded samples were cut into 4-μm-thick sections. The primary antibody anti-MEOX2 (1:200, Affinity; China) was applied overnight at 4°C and a secondary antibody (1:200, Cell Signaling Technology, USA) was used to detect the primary antibody. For quantification of angiogenesis, sections were stained with anti-CD31 (1:50, Abcam; China) and images were obtained using a Leica Microsystems slide scanner (SCN 400; Leica, Mannheim, Germany). Three microscopic fields were selected randomly in each IHC sample and examined by blinded investigators. The staining intensity of MEOX2 was defined as follows: 0 (negative), 1 (weakly positive), 2 (moderately positive), or 3 (strongly positive). The protein expression was evaluated according to the percentage of brown areas in the IHC samples: 0 (<10%), 1 (10%–40%), 2 (40%–70%), and 3 (>70%). The IHC score for each field was expressed as a product of the staining intensity and staining extent (range, 0–9). The angiogenesis index was calculated as the average number of CD31-positive vessels in three independent fields at 20× magnification.

### Cell Culture

MCF-7 and SUM159PT breast carcinoma cells and MCF-10A human mammary epithelial cells were cultured according to the recommendations by the American Type Culture Collection (ATCC). MCF-7 and SUM159PT cells were treated with different doses of Cisplatin (DDP, 1 or 5 μM) or Epirubicin (EPI, 1 or 5 μM).

This study was approved by the Ethics Committee of the First Affiliated Hospital of Xi’an Jiaotong University. All procedures performed in studies involving human participants were in accordance with the ethics standards of the institutional and national research committee and with the 1964 Helsinki Declaration and its later amendments or comparable ethics standards. The ethics committee waived the requirement of written informed consent for participation.

### qRT-PCR

To detect the mRNA levels of MEOX2 in the control and treatment groups, real-time PCR was performed. After treatment with DDP or EPI for 24 h, the MEOX2 mRNA in MCF-7 and SUM159PT cells was extracted in each treatment group. Reverse transcription was immediately performed to obtain the corresponding cDNA and the cDNA fragment was amplified by PCR using the following primers (Ding Guo, China):

F: 5’-TCTCACCAGACTGAGGCGATAC-3’

R: 5’-TCCACTTCATCCGCCTGTTTTGG-3’

Quantification real-time PCR was performed to measure the mRNA levels of MEOX2 in the control and treated cells. Quantitative PCR analysis was performed using the Bio-Rad CFX96TM Real-Time PCR detection system and the results were analyzed with CFXTM Manager 3.0 (BioRad, CA, USA). Each experiment was performed three times and standardized to the level of glyceraldehyde 3-phosphate dehydrogenase (GAPDH).

### Survival Analysis for MEOX2

The Kaplan-Meier plotter (http://kmplot.com/analysis/) can analyze the relationship between gene expression and survival in different types of cancers (16). The prognostic value of MEOX2 in breast cancer was assessed using the breast cancer database. *p* values < 0.05 were considered statistically significant.

### Statistical Analysis

Statistical analysis was performed with GraphPad Prism version 7.00 for Windows (GraphPad Software, La Jolla California USA, www.graphpad). Quantitative data were presented as the mean ± SEM. One-way ANOVA and the Student’s t test was used to examine significant differences between two or multiple groups, respectively. All statistical tests were two-sided. Significance was indicated by *p < 0.05, **p < 0.01, and ***p < 0.001; ns indicated no significance.

## Results

### Identification of DEGs in TCGA and GEO Datasets

**Figure 1:**
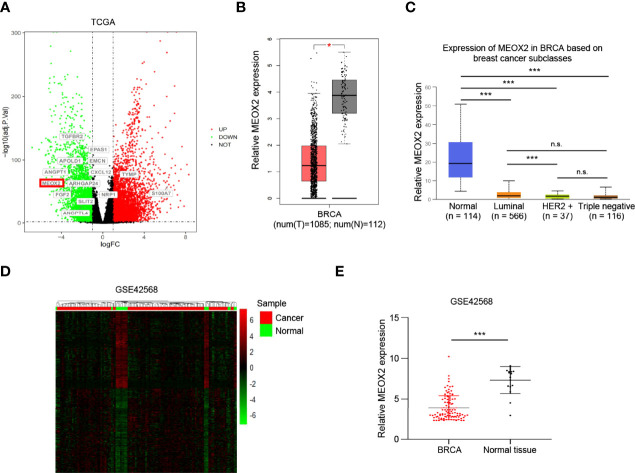
The differentially expressed genes between the healthy controls and the breast cancer patients. **(A)** The volcano plot presents all angiogenesis-related differentially expressed genes in the TCGA dataset (false discovery rate (FDR) <0.05, |log2 fold change (FC)| > 1) (red dots, up-regulated genes; green dots, down-regulated genes). **(B, C)** The MEOX2 gene expression profile across tumor samples and paired normal tissues from the TCGA dataset analyzed with Gene Expression Profiling Interactive Analysis (GEPIA) and the expression in subtype groups analyzed with UALCAN. **(D)** The heat map indicates the differentially expressed genes between the cancer and normal cells in the GEO dataset GSE42568. Each row or column represents one gene or sample, respectively. Green and red bars represent decreased or increased gene expression, respectively. **(E)** Expression of MEOX2 in breast cancer and normal breast tissues in the GEO dataset GSE42568. *p < 0.05, ***p < 0.001, and ns indicated no significance.

The TCGA dataset contains data for 1102 breast cancer cases and 113 normal cases. The DEGs in the dataset were analyzed using the DESeq2 package. In this study, we first performed GO enrichment analysis to obtain related angiogenesis terms with significant differences (**Table S1**). Subsequently, through comparative analysis of the annotations of these genes in **Table S1**, a total of 61 angiogenesis-associated DEGs were identified (using the criteria p < 0.05 and |log FC| > 1); 27 of them were up-regulated and 34 were down-regulated (**Table 1**). A volcano map was plotted to visualize the DEGs (**Figure 1A**). The expression profile of the significantly down-regulated gene MEOX2 was evaluated with Gene Expression Profiling Interactive Analysis (GEPIA) and UALCAN (http://ualcan.path.uab.edu) (17) (**Figures 1B, C**). The results showed that the expression of MEOX2 was lower in breast cancer tissues with different molecular subtype compared to normal tissues. Additionally, the DEGs in the GSE42568 dataset, which contained 104 breast cancer samples and 17 normal samples, were analyzed using the limma package. Using the criteria p < 0.05 and |log FC| > 2, a total of 66 angiogenesis-associated DEGs were identified. The DEGs are shown in a heat map in **Figure 1D**. The expression of MEOX2 in breast cancer samples was also significantly lower than that in normal control samples (**Figure 1E**).

**Table 1:** Sixty-one angiogenesis-related differentially expressed genes (DEGs) identified in the TCGA dataset in breast cancer patients.

DEGs	Gene Name
Up-regulated	KCNJ18, UTS2, TACR3, EDN2, S100A7, CDH2, WT1, PMCH, MYLK2, MYH6, TBX20, ASZ1, CXCL10, GBX2, NKX2-5, CHGA, CARTPT, KCNH2, HRH3, EPO, OLR1, TNNI3, CXCL17, BGN, ESX1, COL1A1, TMPRSS6
Down-regulated	SCN5A, PROX1, TGFBR3, HBB, NTRK2, KCNE1, STAB2, TCF21, EDNRB, APOLD1, ADRB3, TGFBR2, ITGA7, FGF2, PROK1, FGF1, OXTR, PPARG, SOX17, MEOX2, ADRB2, ANGPT1, TACR1, NPR3, MYOCD, ADIPOQ, S1PR1, CAV1, APOB, ATP1A2, NPR1, CAV2, LEPR, EDN3

MEOX2, mesenchyme homeobox 2.

### GO Function Enrichment Analysis

**Figure 2:**
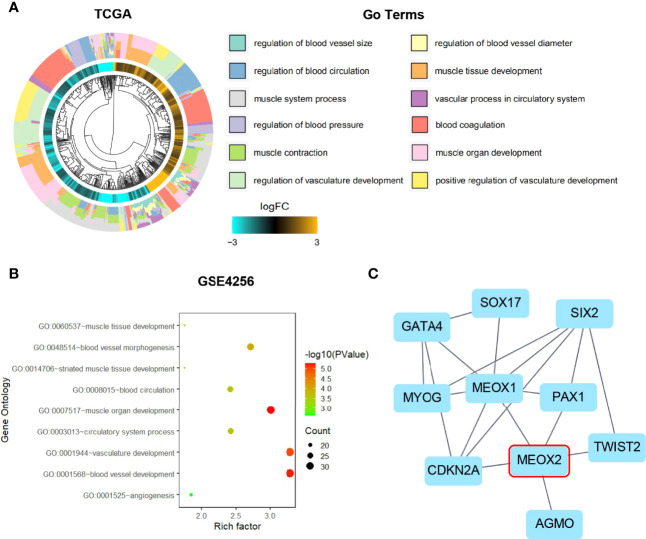
Gene Ontology enrichment and Protein-Protein Interaction analysis. **(A)** GO analysis of the TCGA dataset for the MEOX2 gene. **(B)** GO analysis of the GEO dataset GSE42568. **(C)** The significant module from the PPI network.

To further understand the functional role of MEOX2 (one of the angiogenesis-associated genes), GO functional annotation and pathway enrichment analyses were conducted using the DAVID database. The results suggested that angiogenesis-associated genes were significantly enriched in muscle tissue and organ development, muscle system processes, regulation of blood circulation, regulation of blood vessels, vascular processes in the circulatory system, and regulation of vasculature development (**Figure 2A**). GO enrichment analysis based on the GEO dataset results showed that angiogenesis-associated genes were also significantly enriched in biological processes (BP) including striated muscle tissue development, muscle organ development, circulatory system process, blood circulation, blood vessel morphogenesis, vasculature development, blood vessel development, and angiogenesis (**Figure 2B**).

### PPI Network and Module Analysis

MEOX2 and angiogenesis-associated DEGs were analyzed using the STRING online database and the PPI network was constructed with Cytoscape software. As shown in **Figure 2C**, 10 nodes and 18 edges were identified for MEOX2-associated DEGs. To investigate significant clustering modules in the PPI network, we conducted module analysis using the Cytoscape app MCODE (**Figure 2C**). Further analysis showed that MEOX2, MEOX1, and PAX1 were mainly associated with cellular biosynthetic processes, tissue development, negative regulation of developmental processes, circulatory system development, and the regulation of angiogenesis.

### Expression Level of MEOX2 in Breast Cancer With Drug Treatment in GEO Dataset

**Figure 3:**
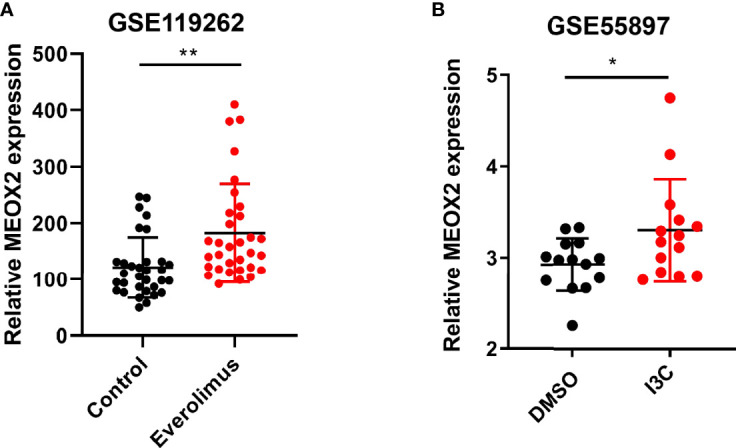
Expression level of MEOX2 is increased in breast cancer with drug treatment in GEO dataset. **(A)** Treatment of breast cancer patients with the mTOR inhibitor everolimus in the GSE119262 dataset. **(B)** Breast cancer cells treated with I3C in the GSE55897 dataset. *p < 0.05, **p < 0.01.

It has been reported that the MEOX homeodomain transcription factor can reduce the expression of angiogenesis-related genes in endothelial cells (18). We found that MEOX2 expression was low in breast cancer and our findings suggested that MEOX2 may repress the occurrence of tumors by inhibiting angiogenesis. Surprisingly, when breast cancer patients were treated with the anti-tumor drug everolimus, the expression of MEOX2 was significantly up-regulated (**Figure 3A**). Moreover, we found that the expression of MEOX2 in breast cancer cells after I3C treatment was significantly higher than that in the dimethyl sulfoxide (DMSO) vehicle group included in the GEO dataset (**Figure 3B**).

### The MEOX2 Expression Was Low in Breast Cancer Tissues and Associated With *Angiogenesis*

**Figure 4:**
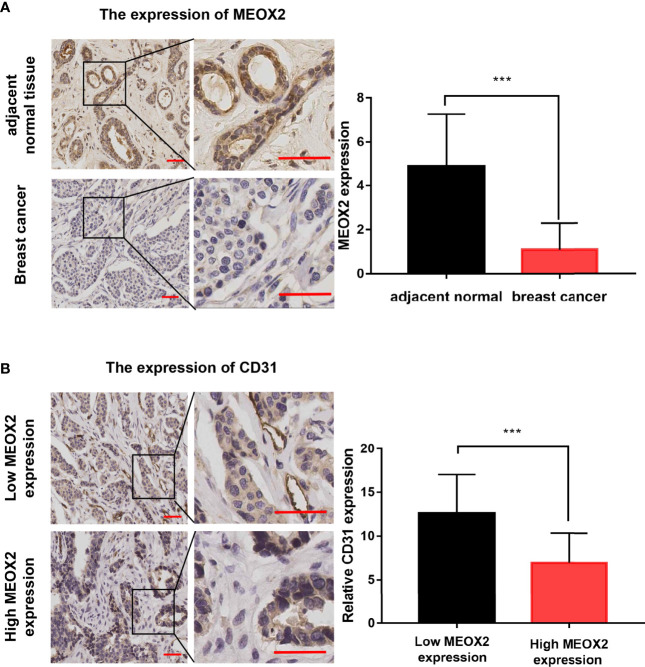
MEOX2 and CD31 expression detected with IHC **(A)** MEOX2 expression in breast cancer tissues and adjacent normal tissues. Scale bar: 50 μm. Quantification of IHC scores (addition of intensity score and positive signal area) (n=20) **(B)** CD31 expression in low MEOX2 group and high MEOX2 group. Scale bar: 50 μm. Quantification of CD31-positive vessels (n=20). ***p < 0.001.

To further investigate the MEXO2 expression in BRCA tissues, 20 breast cancer tissues and 20 adjacent normal tissues were evaluated with immunochemical staining to detect the expression of MEOX2. The results showed that MEOX2 expression in breast cancer tissues was significantly lower than that in adjacent normal tissues (**Figure 4A**; *p* < 0.001). The results of the previous analysis also suggested that the expression of MEOX2 was associated with angiogenesis. Therefore, we examined the expression of CD31, a specific marker for neogenetic microvessels, in groups with different levels of MEOX2. The results showed that a relatively low level of MEOX2 indicated a densely vascularized tissue (**Figure 4B**; *p* < 0.05). Thus, we confirmed that MEOX2 expression was low in breast cancer tissues and was associated with angiogenesis.

### MEOX2 Expression Was Low in Breast Cancer Cells and Elevated by DDP and EPI

**Figure 5:**
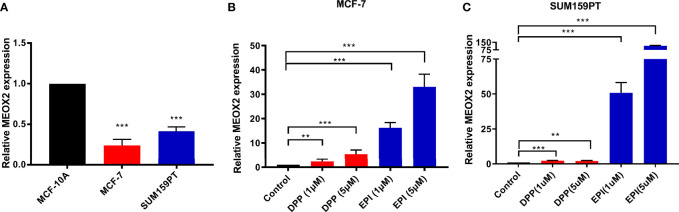
MEOX2 mRNA expression detected with real-time PCR **(A)** in MCF-10A and MCF-7 cells; **(B)** in MCF-7 cells treated with DDP (1 or 5 μM) or EPI (1 or 5 μM) for 24 h; **(C)** in SUM 159PT cells treated with DDP (1 or 5 μM) or EPI (1 or 5 μM) for 24 h. Quantitative data are indicated as mean ± SEM. **p < 0.01; ***p < 0.001).

Next, the expression level of MEOX2 was assessed in breast carcinoma cell lines (MCF-7 and SUM159PT) and the normal human mammary epithelial cell line, MCF-10A. The results revealed that MEOX2 mRNA expression levels in MCF-7 and SUM159PT cells were significantly lower than those in MCF-10A (**Figure 5A**, *p* < 0.001). However, when MCF-7 and SUM159PT cells were treated with DDP (1 and 5 μM) or EPI (1 and 5 μM), the mRNA levels of MEOX2 showed an obvious upward trend (**Figures 5B, C**, *p* < 0.001). This finding confirmed the previous analysis with the GEO database.

### Kaplan–Meier Survival Analysis

**Figure 6:**
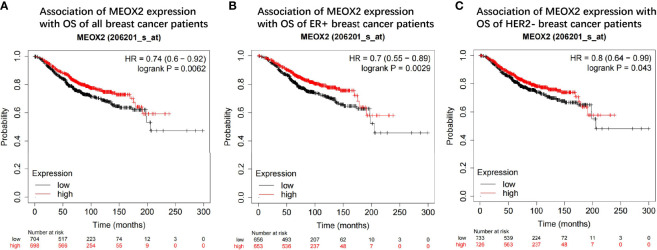
Prognostic value of the MEOX2 gene in breast cancer tissue in KM plotter **(A)** OS of all breast cancer patients; **(B)** OS of ER+ group; **(C)** OS of HER2- group.

The prognostic value of the MEOX2 gene was assessed in the Kaplan-Meier plotter online database by analyzing the overall data for breast cancer patients. The hazard ratios (HRs) and *p* values (log-rank test) showed that high expression of MEOX2 was significantly correlated with better survival in breast cancer patients (**Figure 6A**). The length of survival in the upper quartile of the high expression cohort was 121.2 months, which was significantly higher than the 80.65-month survival in the low expression cohort. In addition, subtype analysis showed that high expression of MEOX2 was significantly correlated with better survival in the estrogen receptor (ER)-positive group and human epidermal growth factor receptor 2 (HER2)-negative group (**Figures 6B, C**).

## Discussion

Breast cancer is the most commonly diagnosed cancer among women and has the second highest mortality rate in women-related cancers (19). Although great progress has been made in the diagnosis and treatment of breast cancer, the prognosis is still poor due to drug resistance or unclear cancer mechanisms (20–23). Angiogenesis is an important cause of breast cancer occurrence and metastasis, and inhibiting the expression of angiogenesis-related genes can effectively inhibit the progression of breast cancer (24–27). However, there is still some confusion regarding the carcinogenic mechanism of angiogenesis. Therefore, specific biomarkers for breast cancer angiogenesis require further exploration.

In the present study, a total of 61 angiogenesis-associated DEGs were identified from the TCGA database for breast cancer. Further analysis of the DEGs with GEPIA and UALCAN revealed that MEOX2 was significantly down-regulated and had different expression levels in different breast cancer subtypes. GO functional annotation analyses showed that MEOX2 was significantly enriched in the regulation of blood circulation, the regulation of blood vessels, vascular processes in the circulatory system, and the regulation of vasculature development. To further confirm MEOX2 expression and function in the GEO database, the GSE42568 dataset was analyzed, and the results were consistent with the above findings.

In the DEGs, PPI network analysis showed that MEOX1, paired box gene 2 (PAX2), cyclin-dependent kinase inhibitor 2A (CDKN2A), and Twist-related protein 2 gene (TWIST2) are closely related to MEOX2. MEOX1 is a member of the transcription factor mesenchyme homeobox and mediates smooth muscle cell differentiation, which is important for vasculogenesis and angiogenesis (28). MEOX1 can regulate the activation of chemokines to define the induction of hematopoietic stem cells (HSCs) (29). The transcription factor PAX2 is involved in tumor angiogenesis (30, 31). The tumor suppressor gene CDKN2A was up-regulated when tumors were treated with inhibitors of angiogenesis (32–35). The binding of TWIST2 to vascular endothelial cadherin (VE-cadherin) promoted vasculogenic mimicry (VM) formation in cancer cells (36, 37). Taken together, these data indicate that MEOX1, PAX2, CDKN2A, and TWIST2 are involved in angiogenesis, which supports our findings.

It has been reported that MEOX2 can reduce the expression of angiogenesis-related genes in endothelial cells (18, 38, 39). Reduced expression of MEOX2 is associated with poor prognosis in hepatocellular carcinoma and laryngeal cancer (40, 41), but there are few reported studies on breast cancer. Using the GEO dataset, we found that the expression of MEOX2 presented an increasing trend both when breast cancer patients were treated with anti-tumor drugs and when breast cancer cells were treated with anti-carcinogenic compounds. We confirmed these findings by IHC staining and real-time PCR. Our experiments showed that MEOX2 expression in breast cancer tissues was significantly lower than that in adjacent normal tissues. The number of CD31-positive vessels in the MEOX2 negative group was significantly higher than that in the MEOX2 positive group. The levels of MEOX2 mRNA in MCF-7 and SUM159PT cells treated with different doses of DDP or EPI were up-regulated. Therefore, MEOX2 might repress tumor development by inhibiting angiogenesis. The Kaplan–Meier plotter online database showed that high expression of MEOX2 was significantly correlated with better survival in breast cancer patients, and the same conclusion was obtained in ER-positive and HER2-negative subgroups. The specific mechanism of MEOX2 stills requires further study.

## Conclusions

To summarize, we conducted bioinformatics analyses of the angiogenesis-related DEGs in breast cancer and identified the significantly down-regulated gene MEOX2 based on the TCGA and GEO databases. Further analysis and experiments revealed that MEOX2 may be related to the prognosis and treatment of breast cancer. Our research indicates that MEOX2 can be used as a novel biomarker for breast cancer diagnosis and clinical therapy.

## Data Availability Statement

The raw data supporting the conclusions of this article will be made available by the authors, without undue reservation.

## Ethics Statement

The studies involving human participants were reviewed and approved by Ethics Committee of the First Affiliated Hospital of Xi’an Jiaotong University. The ethics committee waived the requirement of written informed consent for participation.

## Author Contributions

HW, YT, XY, and PL conceived and designed the research; HW, YT, and WW collected data and conducted the research; HW, SH, and PL analyzed and interpreted data; HW, YT, and PH wrote the initial paper; HW, JZ, and PL revised the paper; HW and PL had primary responsibility for the final content. All authors read and approved the final manuscript.

## Funding

This work was financially supported by funds from the Health Research Foundation of Shaanxi Province (No. 2018D029).

## Conflict of Interest

The authors declare that the research was conducted in the absence of any commercial or financial relationships that could be construed as a potential conflict of interest.

## Publisher’s Note

All claims expressed in this article are solely those of the authors and do not necessarily represent those of their affiliated organizations, or those of the publisher, the editors and the reviewers. Any product that may be evaluated in this article, or claim that may be made by its manufacturer, is not guaranteed or endorsed by the publisher.
