# Supplementary material for: A Crucial Angiogenesis-Associated Gene MEOX2 Could Be a Promising Biomarker Candidate for Breast Cancer
Source: Front Oncol. 2022 May 9;12:759300. doi: 10.3389/fonc.2022.759300 (PMC9124839; doi:10.3389/fonc.2022.759300)
Supplement: Supplementary file 1 [file Table_1.docx]

**Table S1.** The significant gene ontology enrichment terms of angiogenesis-associated DEGs in TCGA dataset.

| Category | Term | Count | FDR | Genes |
| --- | --- | --- | --- | --- |
| BP  BP  BP  BP  BP | GO:0003013~circulatory system process  GO:0008015~blood circulation  GO:0048514~blood vessel morphogenesis  GO:0001944~vasculature development  GO:0001568~blood vessel development | 34  34  27  31  30 | 9.47E-05  9.47E-05  5.61E-03  5.95E-03  6.96E-03 | EDN3, CAV2, KCNJ18, UTS2, CAV1, TACR3, EDN2, TACR1, TBX20, PPARG, ASZ1, OXTR, CXCL10, ADRB3, EDNRB, HRH3, KCNE1, HBB, SCN5A, EPO, OLR1, PMCH, MYLK2, NPR1, ATP1A2, MYH6, NPR3, TNNI3, ADIPOQ, CHGA, ADRB2, **MEOX2**, CARTPT, KCNH2  EDN3, CAV2, KCNJ18, UTS2, CAV1, TACR3, EDN2, TACR1, TBX20, PPARG, ASZ1, OXTR, CXCL10, ADRB3, EDNRB, HRH3, KCNE1, HBB, SCN5A, EPO, OLR1, PMCH, MYLK2, NPR1, ATP1A2, MYH6, NPR3, TNNI3, ADIPOQ, CHGA, ADRB2, **MEOX2**, CARTPT, KCNH2  CAV1, S100A7, LEPR, TBX20, CDH2, WT1, APOB, S1PR1, MYOCD, GBX2, ANGPT1, SOX17, FGF1, NKX2-5, FGF2, TGFBR2, APOLD1, STAB2, TNNI3, TMPRSS6, PROX1, CXCL17, BGN, **MEOX2**, PROK1, ITGA7, NTRK2  CAV1, S100A7, LEPR, TBX20, CDH2, WT1, TCF21, APOB, S1PR1, MYOCD, GBX2, ANGPT1, SOX17, FGF1, NKX2-5, FGF2, TGFBR2, APOLD1, STAB2, ESX1, TNNI3, PROX1, TMPRSS6, CXCL17, BGN, **MEOX2**, PROK1, NTRK2, ITGA7, TGFBR3, COL1A1  CAV1, S100A7, LEPR, TBX20, CDH2, WT1, APOB, S1PR1, MYOCD, GBX2, ANGPT1, SOX17, FGF1, NKX2-5, FGF2, TGFBR2, APOLD1, STAB2, ESX1, TNNI3, PROX1, TMPRSS6, CXCL17, BGN, **MEOX2**, PROK1, NTRK2, ITGA7, TGFBR3, COL1A1 |
